# Bicyclol Regulates Hepatic Gluconeogenesis in Rats with Type 2 Diabetes and Non-alcoholic Fatty Liver Disease by Inhibiting Inflammation

**DOI:** 10.3389/fphar.2021.644129

**Published:** 2021-05-21

**Authors:** Hongxue Li, Qian Xu, Chengye Xu, Yuxin Hu, Xingyang Yu, Kangqi Zhao, Mingqing Li, Meng Li, Junfang Xu, Hongyu Kuang

**Affiliations:** First Affiliated Hospital of Harbin Medical University, Harbin, China

**Keywords:** bicyclol, T2DM, NAFLD, inflammation, hepatic gluconeogenesis

## Abstract

Hepatic gluconeogenesis plays an important role in maintaining the body’s glucose metabolism homeostasis. Non-alcoholic fatty liver disease (NAFLD) is the most common cause of chronic liver diseases, when combined with type 2 diabetes mellitus (T2DM), it can cause severe glucose metabolism disorders. Studies have confirmed that chronic liver inflammatory lesions are the basis of T2DM combined with NAFLD (T2DM–NAFLD), inhibiting liver inflammation can improve glucose metabolism disorders. It is essential to explore safe and effective drugs to inhibit liver inflammation to improve the body’s glucose metabolism disorders. Bicyclol is a biphenyl derivative that has anti-oxidative and anti-inflammatory properties. In the present study, the hepatoprotective effects and underlying mechanisms of bicyclol in T2DM–NAFLD were investigated, and T2DM–NAFLD with/without bicyclol treatment models were established. The results revealed that bicyclol alleviated fasting blood glucose, serum transaminase levels, insulin resistance, hepatic adipogenesis, lipid accumulation and markedly reduced T2DM–NAFLD rat histological alterations of livers. Not only that, bicyclol markedly attenuated T2DM–NAFLD induced production of inflammation factors (IL-1β and TNF-α). Moreover, bicyclol suppressed the expression of insulin/gluconeogenesis signaling pathway (Akt, PGC-1α and PEPCK). These findings suggested that bicyclol might be a potentially effective drug for the treatment of T2DM–NAFLD and other metabolic disorders.

## Introduction

Glucose homeostasis is the basis of maintaining the body’s biological functions, which mainly depends on the cooperation of insulin-secretory capacity of pancreas, endogenous glucose output and utilization of peripheral tissues (Kang et al., 2014). To carry through its function, endogenous glucose output which mainly occurred in liver plays the major role. In fasting conditions, the liver takes over 90% of the endogenous glucose output through hepatic gluconeogenesis which is great significance for maintaining glucose homeostasis (Landau et al., 1996). Therefore, the liver plays a central role in the control of glucose production.

NAFLD is now recognized as the most prevalent chronic liver disease worldwide, with a global prevalence of 25.2%, which has become the main cause of morbidity and mortality of liver-related diseases (Younossi et al., 2019). As we all known, NAFLD is strongly correlated with T2DM, the global prevalence of T2DM as 22.5% among patients with NAFLD. On the other hand, approximately 45% of NAFLD patients have diabetes (Younossi et al., 2016). The initial accumulation of fat followed by subsequent inflammation is central to the development of NAFLD liver damage (Than and Newsome, 2015). When NAFLD combined with T2DM, the liver damage accelerates and the risk of death is significantly increased. At the same time, the body’s glucose homeostasis is imbalanced, leading to further aggravation of glucose metabolism disorders. It is well established that insulin resistance, obesity, chronic inflammation are common pathogenic factors of T2DM–NAFLD. Inflammation-induced IR is considered to be a typical feature of T2DM and the basis of NAFLD. Numerous studies have shown that inhibiting inflammation can significantly improve liver damage in T2DM with NAFLD (Giribabu et al., 2018; Leite et al., 2019). At the same time, it has been found that a variety of drugs can improve liver inflammatory lesions and bring benefits to T2DM–NAFLD induced glucose metabolism disorders (Park et al., 2013; De Souza et al., 2015). It is essential to explore safe and effective drugs for the treatment of chronic liver inflammation and improve the body’s glucose metabolism disorders.

Bicyclol (4,4′-dimethoxy-5,6,5′,6′-dimethylenedioxy-2-hydroxymethyl-2′-carbonyl biphenyl) is an approved drug in China, which is widely used in anti-hepatitis, anti-liver fibrosis and anti-liver injury (Li and Gt, 2004). Moreover, growing evidence indicates that bicyclol has multiple biological effects that may be helpful in the treatment or prevention of T2DM–NAFLD (Han et al., 2014). Hepatic gluconeogenesis is a key metabolic mechanism of hyperglycemia in the body, and the increased hepatic glucose production is a major cause of glucose metabolism disorders in T2DM–NAFLD. Our study tried to analyze the effects of bicyclol on hepatic gluconeogenesis in T2DM–NAFLD rats from the perspective of inhibiting liver inflammation, in order to provide safe and efficient therapeutic targets for T2DM–NAFLD and other metabolic diseases.

## Materials and Methods

### Drugs and Chemicals

Bicyclol was purchased from Beijing Union Pharmaceutical Factory (Beijing, China). Streptozotocin (STZ) was purchased from Sigma (St. Louis, MO, United States). Rat Interleukin-1β (IL-1β), tumor necrosis factor-α (TNF-α) and phosphoenolpyruvate carboxy kinase (PEPCK) enzyme-linked immunosorbent assay kits were purchased from Bosterbio (Wuhan, China). Total cholesterol (TC), triglyceride (TG), alanine aminotransferase (ALT) and glutamic-oxaloacetic transaminase (AST) kits were purchased from Nanjing Jiancheng Bioengineering Institute (Nanjing, China). Serine protein kinase (Akt), phosphorylates serine protein kinase (*p*-Akt), peroxisome proliferator-activated receptor γ coactiva-tor-1α (PGC-1α), IL-1β and TNF-α antibodies were purchased from Wanleibio (Shenyang, China). PEPCK antibody was purchased from Proteintech Group (Chicago, United States). BCA kit was purchased from Beyotime (China).

### Animal Models and Treatment

**FIGURE 1:**
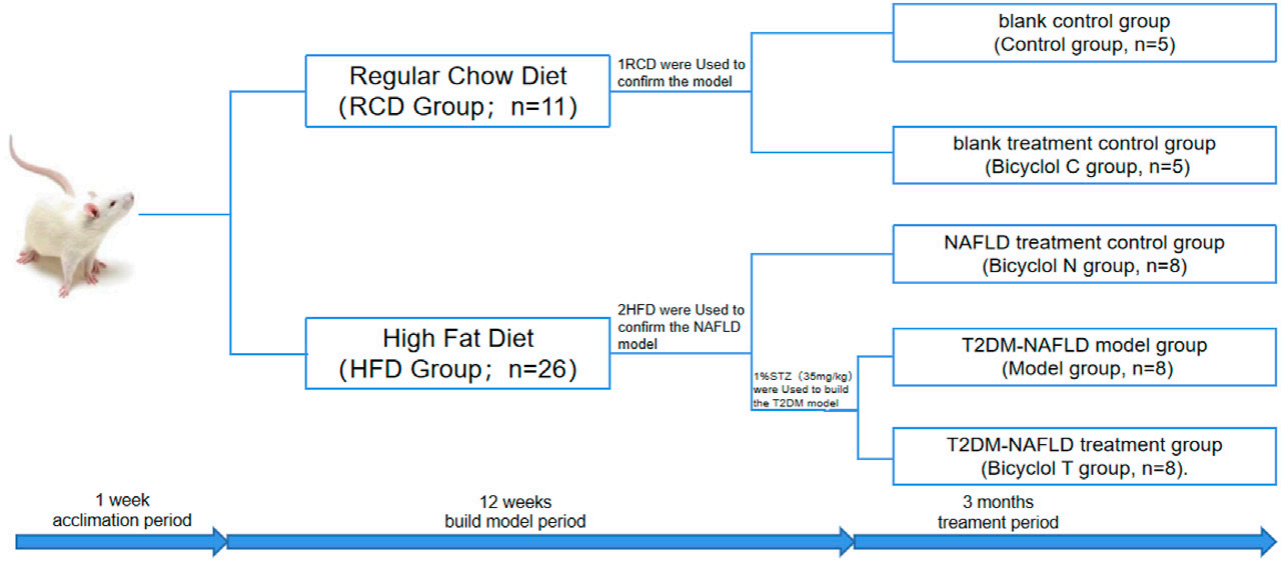
Schematic diagram of experiment grouping.

Thirty seven male Sprague-Dawley rats (6 weeks old, 120–130 g) were purchased from Heilongjiang University Of Chinese Medicine (Harbin, China). The rats were housed in a temperature-controlled room (22 ± 2°C) with a 12°h light/dark cycle and a relative humidity of 40–70%. They were given free access to food and water and the body weight of the rats were determined weekly. The rats were randomly assigned to the following five groups as shown in Figure 1. The study was approved by the Ethics Committee of The First Affiliated Hospital of Harbin Medical University and conformed to the principles of the Declaration of Helsinki. All animal procedures were conducted in accordance with the National Institutes of Health’s Guide for the Care and Use of Laboratory Animals (NIH Publications Number 8023, Revised 1978).

After acclimation period of 1 week, the rats were randomly divided into two groups, the regular chow diet group (RCD group, *n* = 11) and the high-fat diet (the composition was as follows: 10% lard, 20% sucrose, and 70% regular chow diet) group (HFD group, *n* = 26). After 10 weeks, one rat in group RCD and two rats in group HFD were used to confirm the model. At the same time, The group of RCD were randomly divided into blank control group (Control group, *n* = 5) and blank treatment group (Bicyclol C group, *n* = 5). The HFD group were randomly divided into NAFLD treatment group (Bicyclol N group, *n* = 8), T2DM–NAFLD model control group (Model group, *n* = 8), and T2DM–NAFLD treatment group (Bicyclol T group, *n* = 8). Model group and Bicyclol T group were given an intraperitoneal (i.p.) injection of 1% streptozotocin (STZ) at a dose of 35 mg/kg 72 h after STZ injection, fasting blood glucose (FBG) were measured by using the blood glucose meter for two days continuously, and FBG ≥7.8 mmol/L was used to indicate a successful diabetes model.

The 12th weeks after modeling, the Control group and the Model group were given 5 ml purified water daily, and the bicyclol-treated groups were given bicyclol at a dose of 300 mg/kg/day (i.g.) for 3 months. At the end of the experiment, five rats remained in each group and all 25 animals were anesthetized with 2% pentobarbital at a dose of 60 mg/kg (i.p.), blood was collected by abdominal aorta and centrifuged, serum was obtained for biochemical tests. The liver was rapidly removed, weighed and sectioned, and appropriate storage for each analysis was conducted. The wet weight of the liver was measured to calculate the liver index using the following equation: Liver index = liver wet weight/body weight × 100%.

### Measurement of Serum Biochemical Parameters

FBG levels were measured by using a blood glucose meter (BAYER, Germany). Serum insulin levels were determined by using a semi-auto analyzer AIA-1800 (Tosoh Corporation, Japan). The homeostasis model of assessment-insulin resistance (HOMA-IR) was calculated as follows: HOMA-IR=(FBG × FINS)/22.5. Serum TC, TG, ALT, and AST were measured by a commercially available kit according to manufacturer instructions.

### Histopathology and Ultrastructural Changes

4% paraformaldehyde-fixed liver tissue were embedded in paraffin, sectioned at a thickness of 4 μm and stained with hematoxylin and eosin. Histological evaluation of the liver sections were observed under an optical microscope. At the same time, other liver tissues were fixed with 2.5% glutaraldehyde, and then tissues were embedded, sectioned, and double-stained with 3% uranyl acetate-lead citrate, ultrastructural changes were captured with a Transmission Electron Microscopy (JEOL, Tokyo, Japan).

### Elisa Analysis

Levels of TNF-α, IL-1β and PEPCK in serum were evaluated by using ELISA kits, according to the manufacturer’s instructions.

### Immunohistochemistry Analysis

4% paraformaldehyde-fixed and paraffin embedded liver sections were mounted on glass slides, the liver tissue was deparaffinized, renovated by citric acid (pH 6.0), incubated in 3% H_2_O_2_ for 25 min and blocked with 10% Normal Goat Serum 30 min. Then, slides were incubated, respectively, with anti-*p*-Akt, anti-PGC-1α, anti-TNF-α, anti-IL-1β, and anti-PEPCK for over-night at 4°C. Incubation with secondary antibodies for 50 min. After a washing step with PBS, 3,3′-diaminobenzidine was used for 3 min and counterstained with hematoxylin. Images were taken at × 400 original magnification (Nikon, Japan).

### Western Blot

Total protein of the rat liver was extracted by RIPA lysis buffer with 1% protease inhibitor and 10% phosphatase inhibitor. The concentration of protein was measured using the BCA kit and adjusted using RIPA lysis buffer. Then, the protein were separated through SDS-PAGE and transferred onto PVDF membranes. After blocking with 5% skimmed milk for 1 h, membranes were then incubated in antibody overnight at 4°C and incubated with the secondary antibody at room temperature for 1 h after being washed with PBST for 3 times. Then, the protein bands were detected with an Odyssey infrared laser scan-imaging instrument (LI-COR), and the images were analyzed using Odyssey Application Software 5.2.5.

### Statistical Analysis

All data were expressed as mean ± S.D. Comparisons between multiple groups were carried out *via* one-way analysis of variance, and statistical analysis was performed by GraphPad Prism eight software. Values with *p* < 0.05 were considered statistically significant.

## Results

### Effects of Bicyclol on Body Weight, Liver Index, Liver Size, and Histopathological Examination in Rats

**FIGURE 2:**
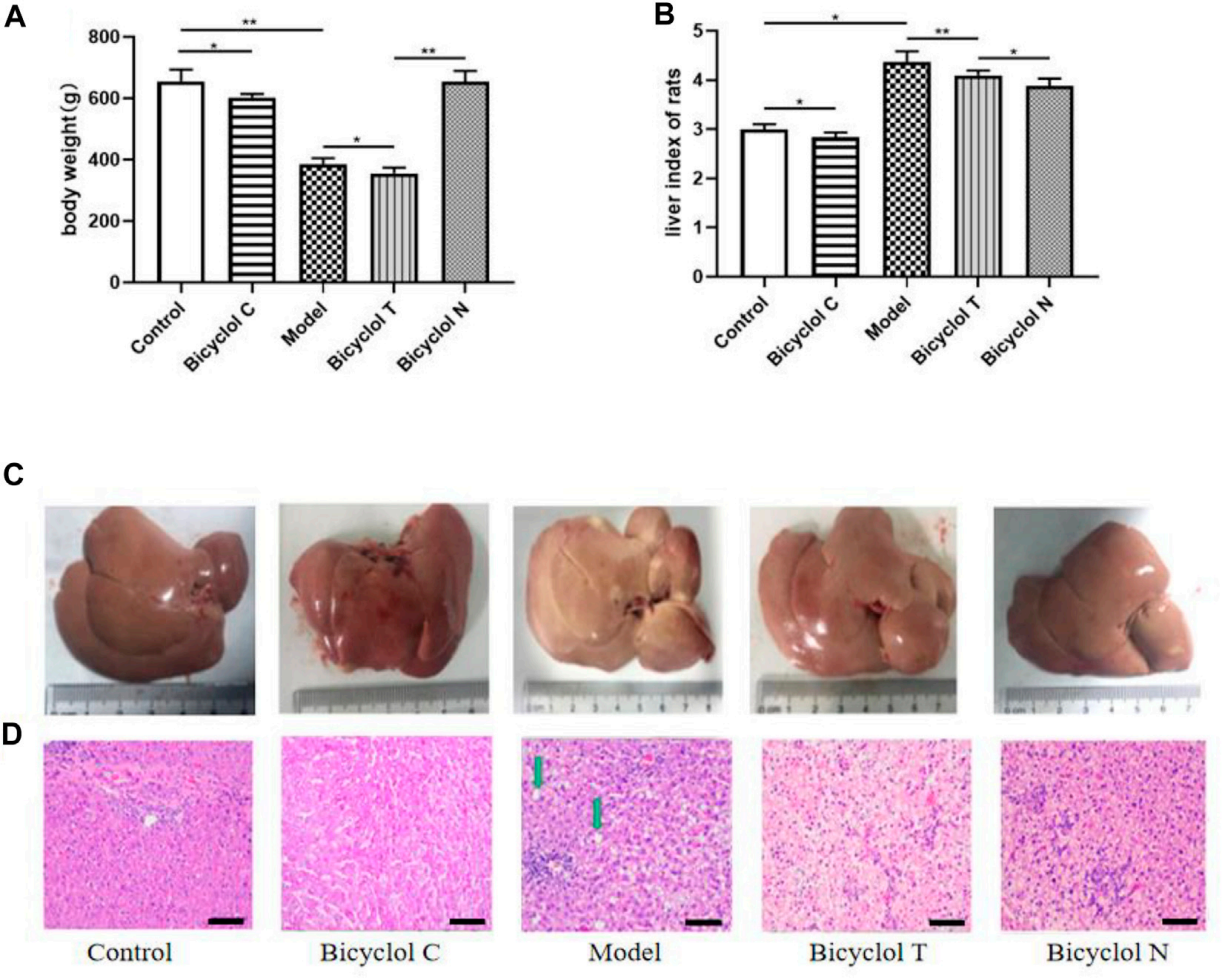
Effect of bicyclol on the body weight, volume of liver and histopathological examination in T2DM–NAFLD rat. **(A)** Body weight. **(B)** Liver index of rats. **(C)** Appearance of the liver. **(D)** Hematoxylin and eosin staining: lipid droplets (green arrow) in livers. Data are presented as means ± SD. *p < 0.05. **p < 0.01.

After 3 months, the body weight and liver index of rats in Model group and Bicyclol C group was significantly decreased, compared with the normal rats in Control group (*p* < 0.05). The body weight gain in group Bicyclol T was lower than that in the Model group (*p* < 0.05) and Bicyclol N group (*p* < 0.05) (Figures 2A,B).

Observed with naked eyes, the liver from Control group was deep red, glossy, moist, and resilient. While in Model group, the liver was enlarged, lost luster and tough texture. Liver injury was mitigated dramatically after bicyclol treatment. Consistent with these findings, the liver size was reduced markedly in bicyclol-treated groups (Figure 2C).

HE stained sections are shown in Figure 2D, in Control and Bicyclol C group, the histology of the liver displayed that a completely normal hepatic parenchyma. However, in the Model group, most of the hepatocytes were swollen and varied in size, the edge was fuzzy, the structure of the liver lobules was unclear and there were a lot of lipid droplets. Treatment with bicyclol prevented the histopathological changes and reduced the liver lesions, in the Bicyclol N group, the histology of the liver displayed a normal structure and some lipid droplets. In the Bicyclol T group, the liver lobules showed a relatively normal structure and a few lipid droplets. According to these results, bicyclol could reduce the body weight and liver index, improve liver and fat accumulation and then.

### Bicyclol Improve FBG, Insulin Sensitivity and Serum Lipid Profile in HFD-Fed Rats

**FIGURE 3:**
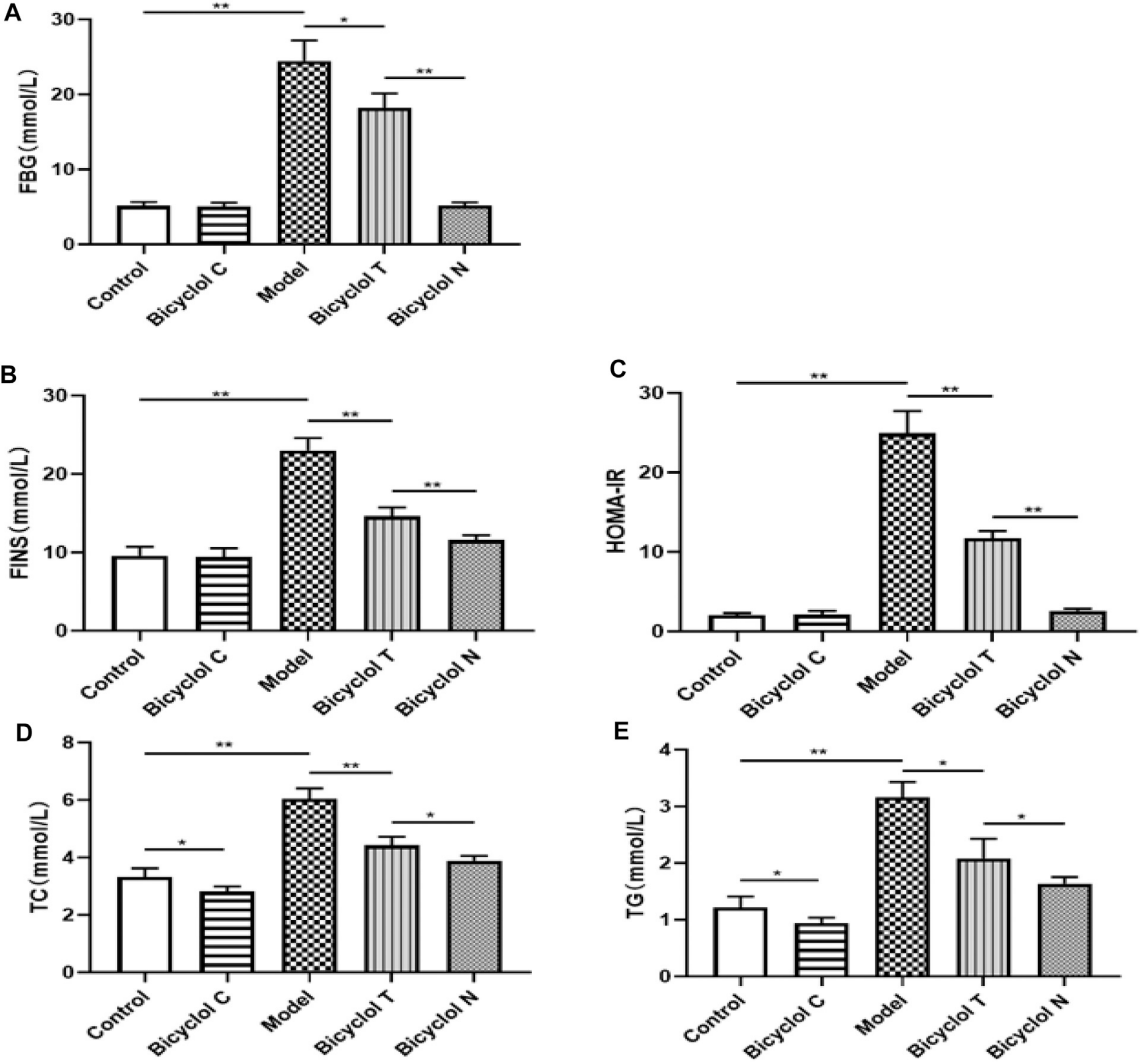
Effect of bicyclol on blood lipid and glucose levels in T2DM–NAFLD rat **(A)** FBG. **(B)** FINS. **(C)** HOMA-IR. **(D)** TC. **(E)** TG. Data are presented as means ± SD. *p < 0.05. **p < 0.01.

The FBG level in Model group was higher than that in Control group (*p* < 0.01). Compared with the Model group, blood glucose were obviously decreased in Bicyclol T group (*p* < 0.05); Compared with Bicyclol N group, the FBG level in Bicyclol T group was obviously increased (Figure 3A). Further research demonstrated that the level of insulin and HOMA-IR in Bicyclol T group was lower than that in Model group (*p* < 0.01) and higher than that in Bicyclol N group (*p* < 0.01) (Figures 3B,C).

Compared with Control group, The TC, TG levels were significantly higher in Model group (*p* < 0.01) and lower in Bicyclol C group (*p* < 0.05); while the TC, TG levels in Bicyclol T group were decreased than Model group (*p* < 0.01, *p* < 0.05) and increased than Bicyclol N group (*p* < 0.05) (Figures 3D,E).The results indicated that bicyclol could decrease the blood glucose and improve lipid metabolism in T2DM–NAFLD rats.

### Bicyclol Attenuates Markers of Liver Damage in HFD-Fed Rats

**FIGURE 4:**
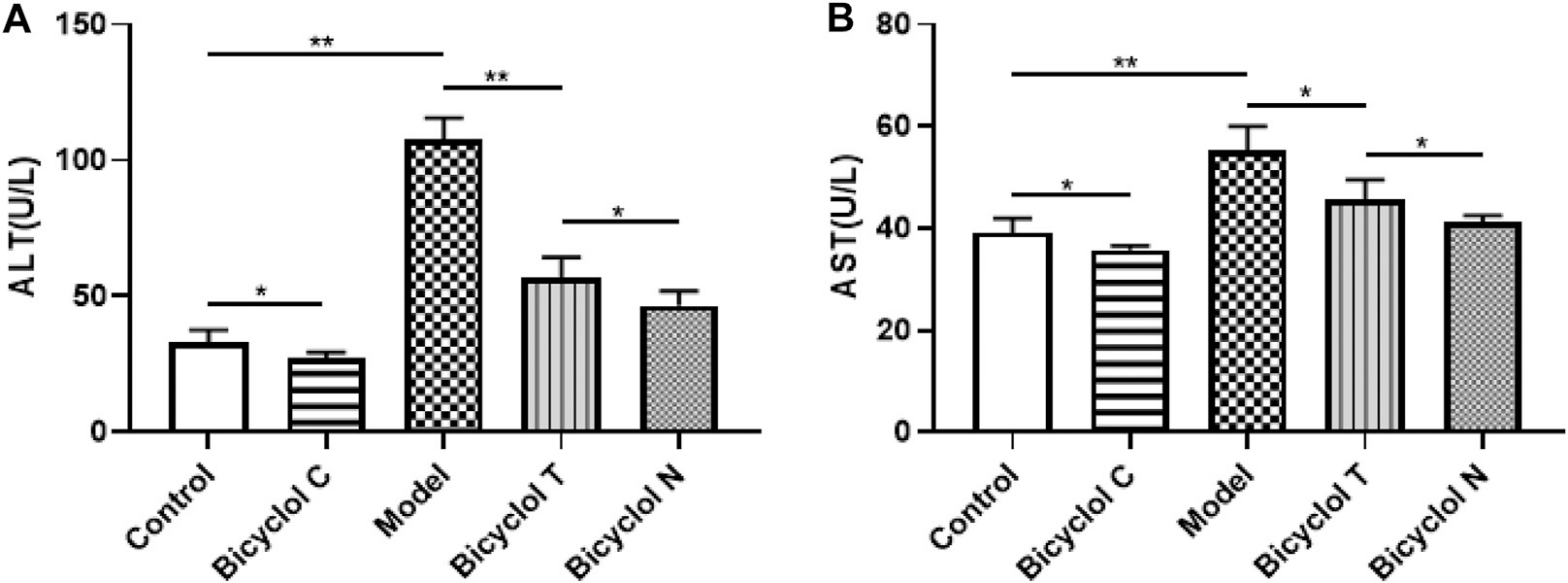
Effect of bicyclol on ALT and AST levels in T2DM–NAFLD rat **(A)** ALT. **(B)** AST. Data are presented as means ± SD. *p < 0.05. **p < 0.01.

As shown in Figure 4, compared with the Control group, the levels of serum ALT and AST were increased significantly in the Model group (*p* < 0.01) and were decreased in Bicyclol C group (*p* < 0.05). While compared with the Model group, the levels of ALT and AST markedly decreased in Bicyclol T group (*p* < 0.01, *p* < 0.05). Compared with the Bicyclol N group, the levels of ALT and AST were increased in Bicyclol T group (*p* < 0.05). The results suggested that bicyclol could improve liver function in T2DM–NAFLD rats.

### Bicyclol Relieved Liver and Serum Inflammation in HFD-Fed Rats

**FIGURE 5:**
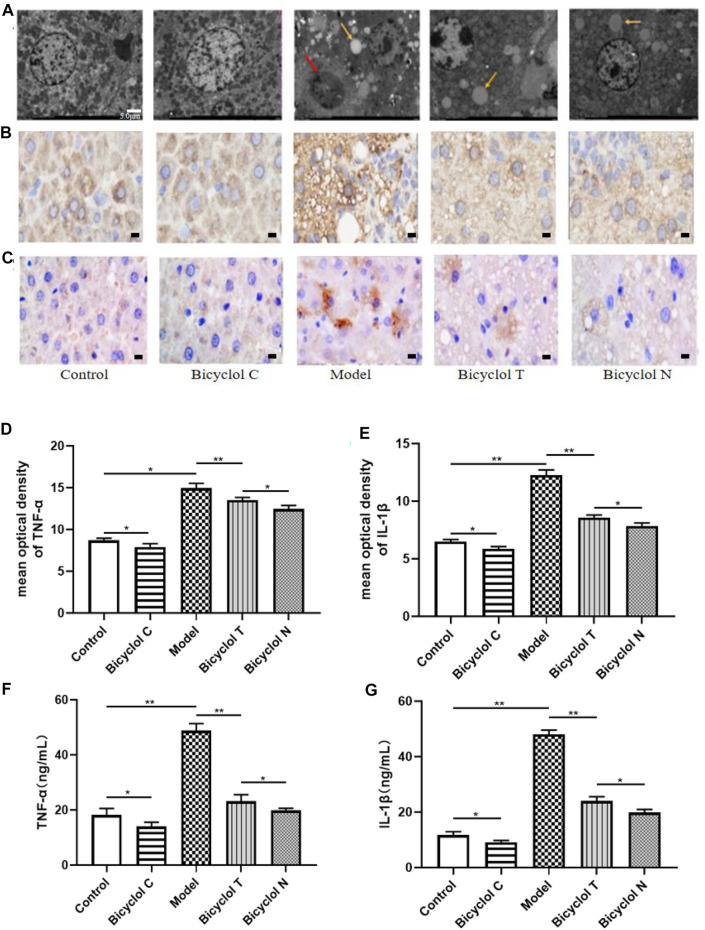
Effect of bicyclol on liver and serum inflammation in T2DM with NAFLD rat **(A)** TEM images (*1.5 k) from TEM showing characteristic inflammasome (red arrow) and lipid droplets (yellow arrow) in livers. **(B)** The expression of TNF-α determined by IHC. **(C)** The expression of IL-1β determined by IHC. **(D)** Mean optical density analysis of TNF-α expression in liver. **(E)** Mean optical density analysis of IL-1β expression in liver. **(F)** The levels of TNF-α in serumv **(G)** The levels of IL-1β in serum. Data are presented as means ± SD. *p < 0.05. **p < 0.01.

As shown in Figure 5A, the arrangement of hepatocytes in Control group and Bicyclol C group was dense and neat, and the size of mitochondria was relatively uniform without swollen. However, in the Model group, hepatocyte nucleus shrank and broken into multiple pieces, mitochondria were arranged disorder with many lipid droplets and inflammasomes. Whereas lipid droplets and inflammasomes decreased significantly after bicyclol treatment.

Elisa kits and immunohistochemistry (IHC) were used to test the levels of the traditional inflammatory factors (TNF-α, IL-1β) in serum and liver. Compared with the Control group, the levels of TNF-α, IL-1β were increased significantly in the Model group (*p* < 0.01) and decreased in the Bicyclol C group (*p* < 0.05). While compared with the Model group, the levels of TNF-α, IL-1β markedly decreased in Bicyclol T group (*p* < 0.01). Compared with the Bicyclol N group, the levels of TNF-α, IL-1β were increased in Bicyclol T group (*p* < 0.05) (Figures 5B–G). The results demonstrated that bicyclol could decrease inflammation of serum and liver in T2DM–NAFLD rats.

### Effects of Bicyclol on the Expression of Akt/PGC-1α

**FIGURE 6:**
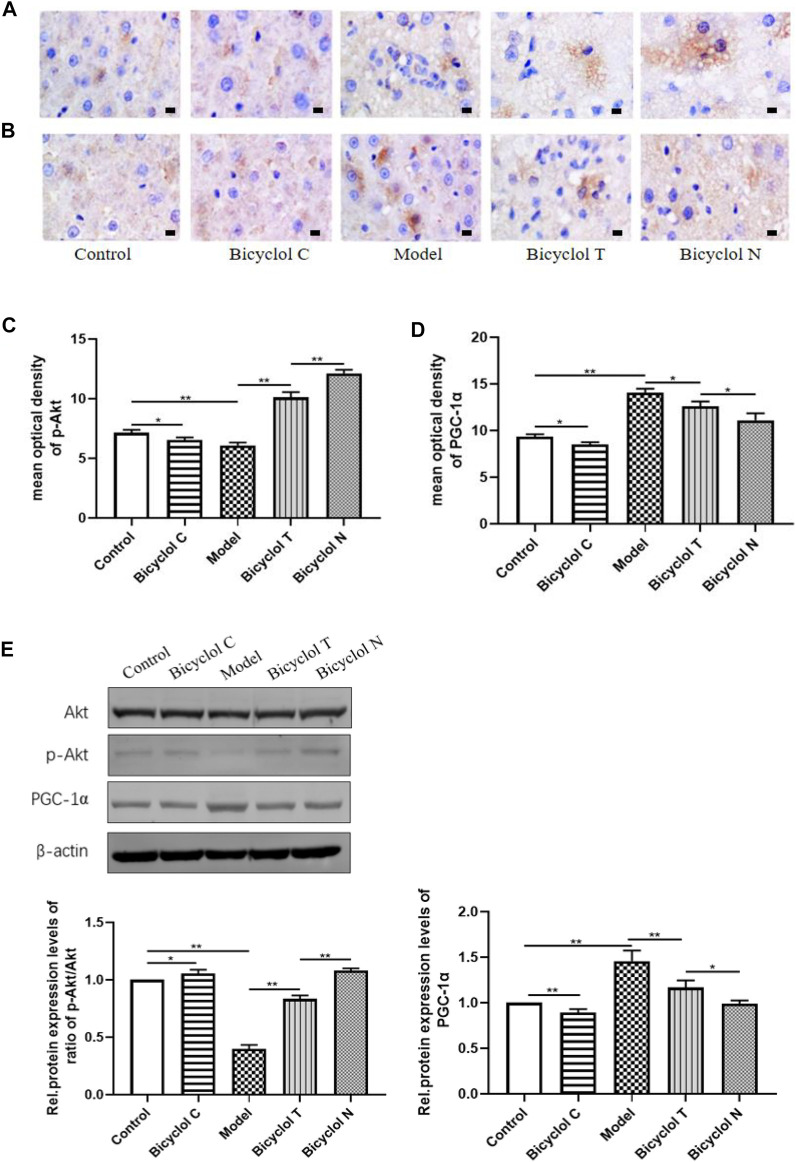
Effect of bicyclol on regulating gene expression of Akt/PGC-1α signaling pathway in T2DM with NAFLD rat **(A)** The expression of *p*-Akt determined by IHC. **(B)** The expression of PGC-1α determined by IHC. **(C)** Mean optical density analysis of *p*-Akt expressiont in liver. **(D)** Mean optical density analysis of PGC-1α expression in liver. **(E)** Western blot analysis of Akt, *p*-Akt, PGC-1α in liver. Data are presented as means ± SD. *p < 0.05. **p < 0.01.

To test whether Akt/PGC-1α pathway involves in the mechanism of T2DM–NAFLD, we examined their expression by immunohistochemistry and western blot. The results indicated that the expression of *p*-Akt was lower in Model group (*p* < 0.01) and higher in Bicyclol C group (*p* < 0.05) than that in Control group. The level of *p*-Akt was higher in Bicyclol T group (*p* < 0.01) than Model group after treatment. While compared with Bicyclol N group, the expression of *p*-Akt was decreased in Bicyclol T group (*p* < 0.01). Immunohistochemistry and western blot also indicated that the expression level of PGC-1α was higher in Model group (*p* < 0.01) and lower in Bicyclol C group (*p* < 0.05) than that in Control group. After treatment, the level of PGC-1α was lower in Bicyclol T group (*p* < 0.05) than Model group. While compared with Bicyclol N group, the expression of PGC-1α was increased in Bicyclol T group (*p* < 0.05) (Figure 6). The results indicated that bicyclol could increase the phosphorylation of Akt, down-regulate protein level of PGC-1α in T2DM–NAFLD rats.

### Increased Hepatic Gluconeogenesis in Liver and Serum from HFD-Fed Rats and Suppressed by Bicyclol

**FIGURE 7:**
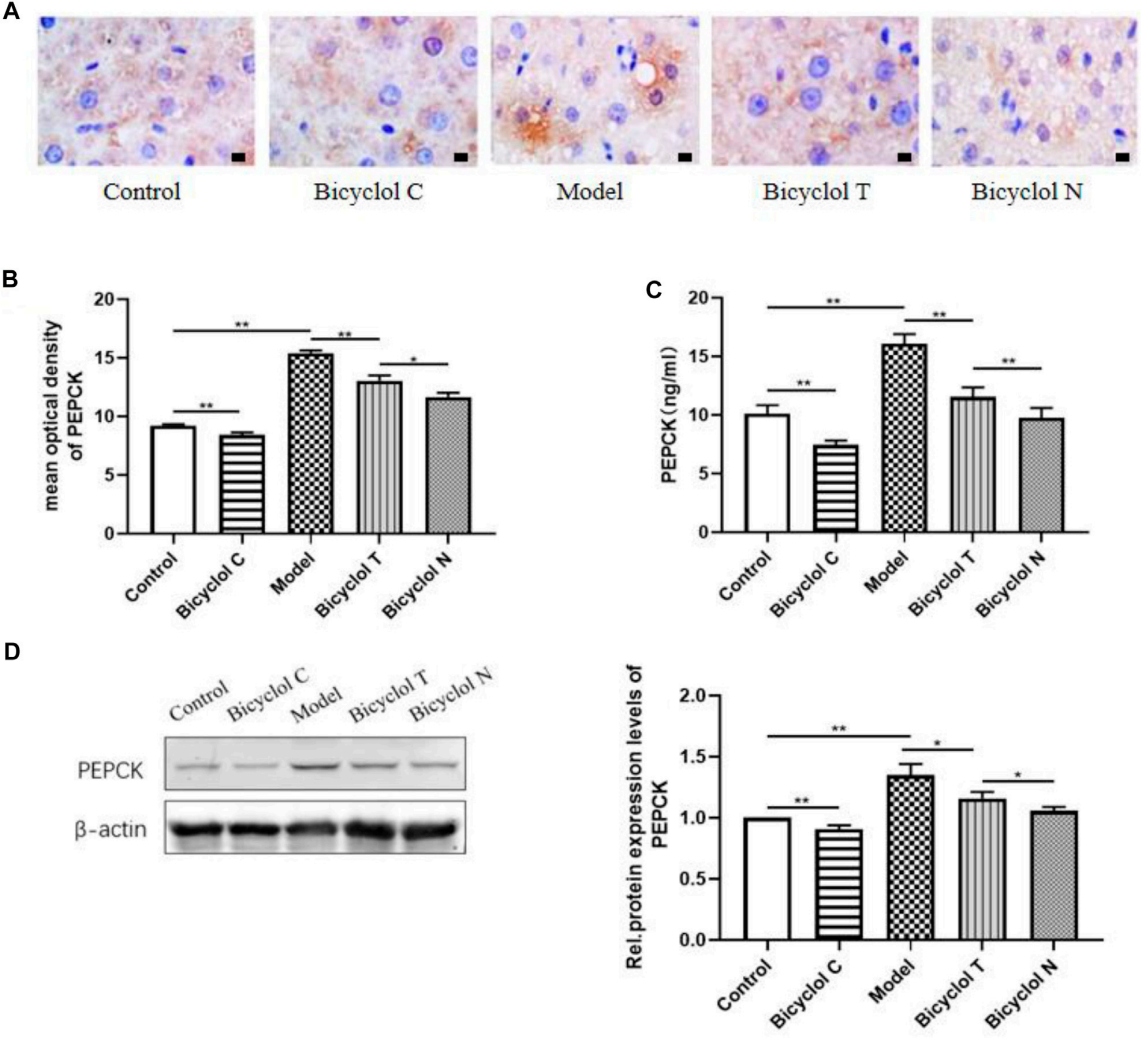
Effect of bicyclol on PEPCK in T2DM with NAFLD rat serum and liver **(A)** the expression of PEPCK determined by IHC. **(B)** Mean optical density analysis of PEPCK expression in liver. **(C)** The level of PEPCK in serum. **(D)** Western blot analysis of PEPCK in liver. Data are presented as means ± SD. *p < 0.05. **p < 0.01.

Elisa kits, IHC and Western blot tested the expression of the gluconeogenesis-related protein (PEPCK) in serum and liver. Compared with the Control group, the expression of PEPCK were increased significantly in the Model group (*p* < 0.01) and decreased in the Bicyclol C group (*p* < 0.01).While compared with the Model group, the expression of PEPCK markedly decreased in Bicyclol T group (*p* < 0.01). Compared with the Bicyclol N group, the expression of PEPCK was increased in Bicyclol T group (*p* < 0.05) (Figure 7). The results demonstrated that bicyclol could decrease hepatic gluconeogenesis in serum and liver in T2DM–NAFLD rats.

## Discussion

Disorder of glucose metabolism is the key cause of hyperglycemia which is often combined with hyperinsulinemia to induce many metabolic diseases (Giugliano et al., 2008). Worldwide, T2DM combined with NAFLD is a common metabolic disease with a chronic hepatitis trait. It is a crucial health issue related to the liver injury, blood glucose disorder and affect the quality of people life (Feneberg and Malfertheiner, 2012). Feeding an HFD is widely used to induce hepatic steatosis in experimental rats, and SD rats appear susceptible to steatohepatitis development when fed an HFD (Takahashi et al., 2012). Here, we used SD rats to establish T2DM–NAFLD model by HFD and STZ (i.p.). Through compared the bicyclol-treated T2DM–NAFLD group and bicyclol-treated NAFLD group, we found that glucose metabolism disorder would take a huge damage to rats. Hepatic gluconeogenesis can maintain the glucose and lipid of cellular or whole-body in a steady state, so it plays a vital part in hepatic glucose metabolism (Gastaldelli et al., 2000; Wu et al., 2018). Convincing evidence indicates that overactivated hepatic gluconeogenesis and lipogenesis contribute to the pathogenesis of metabolic disorders, including DM and NAFLD (Cui et al., 2018; Dong et al., 2019; Li et al., 2020). Therefore, one of the important targets in the treatment of T2DM–NAFLD is to inhibit the glycolipid toxicity caused by gluconeogenesis.

The potential mechanism of the occurrence and progression of T2DM–NAFLD is complex and multifactorial, the traditional theory of “two-hits” has been challenged, and the interaction of various pathogenic factors has been gradually understood (Tilg et al., 2016). At present, many studies have found that hepatic inflammation plays an important role in the development of metabolic disorders caused by T2DM–NAFLD (Morrison and Kleemann, 2015; Dewidar et al., 2020; Gehrke and Schattenberg, 2020). So it is essential to exploring a safe and effective drug to inhibit hepatic inflammation and improve hyperglycemia. It is suggested that bicyclol exhibits anti-inflammatory effect under diverse liver-injury condition (Li and Gt, 2004). In present studies bicyclol were used in healthy rat, NAFLD rat and T2DM–NAFLD rat, compared with healthy-rat and T2DM–NAFLD rat, our work suggested that bicyclol might effectively improve T2DM–NAFLD related symptoms include improve the hepatic insulin signal, suppress enhancement of the hepatic gluconeogenesis and reduce the liver injury through an anti-inflammatory mechanism.

TNF-α and IL-1β, classic inflammatory factors, have the functions of promoting inflammation, regulating cell proliferation, differentiation and apoptosis (Zelova and Hosek, 2013). Several of these cytokines are known to alter fatty acid metabolism by the liver. Our study found that HFD increased the TNF-α and IL-1β levels in serum and liver, similar to the studies by Borowska et al. (2016). Suggesting that the liver inflammation happened in the T2DM–NAFLD rat. After bicyclol-treated, the levels of TNF-α and IL-1β were decreased and the pathological and ultrastructural changes of liver tissue are also weakened, it is demonstrated that bicyclol markedly inhibiting the inflammation in T2DM–NAFLD rat. To the best of our knowledge, the serum levels of the two hepatic marker enzymes (ALT and AST) are correlated with liver injury and diabetes. Herein, our results revealed that bicyclol can improve ALT and AST levels means inhibiting inflammation can reduce liver injury. Inflammation is believed to be an important step in the pathogenesis of IR (Adabimohazab et al., 2016). What’s more, IR and inflammation form a vicious circle, each condition promoting the other and accelerating the development of metabolic disorders in the presence of lipotoxicity (Tanase et al., 2020). IR is typically quite pronounced in T2DM, but those with NAFLD had marked IR, more severe than those with T2DM without NAFLD (Kelley et al., 2003). HOMA-IR is considered a surrogate marker for IR, although the hyperinsulinemic-euglycemic clamp is the gold standard for evaluating IR, it is difficult to apply this method to large-scale epidemiological studies. Thus, HOMA-IR is often used as an indirect indicator of insulin sensitivity. In our study, bicyclol can markedly decreased the level of HOMA-IR, our findings revealed that bicyclol can Significantly inhibit the occurrence of IR.

When IR occurs, the ability of insulin to inhibit hepatic glucose is decreased, hepatic gluconeogenesis is increased, may be caused by overnutrition, decreased portal vein insulin/glucagon ratios and/or impaired insulin signaling (Roden, 2008). Therefore, we can reasonably assume that there is a defect in the insulin signaling pathway in T2DM–NAFLD rat with high IR status. Over the last couple of decades, accumulating evidence has shown Akt as a central regulator of insulin action, and Akt plays an essential role in promoting glucose uptake and cell growth, survival, and proliferation in response to cytokines and growth factors (Yudushkin, 2019). In this study, the level of *p*-Akt/Akt in T2DM–NAFLD rat was decreased, it’s shown the activation of Akt and the insulin signaling pathway has been broken. The pathway diversification of hepatic insulin signaling appears to occur largely distal to Akt activation. Akt substrates include GSK3, FOXO1, PGC-1α and mTORC1, which control anabolic program upregulating gluconeogenic genes, lipogenic gene expression and protein synthesis in turn (Petersen and Shulman, 2018). Active, PGC-1α, a key regulator of glucose production in the liver, can upregulate hepatic gluconeogenic gene expression, such as PEPCK, selective inhibition of PGC-1α potentially reduced hepatic glucose production and ameliorated hepatic insulin resistance (Fang et al., 2019). What’s more, Akt/PGC-1α inhibition has even been shown to reduce gluconeogenic gene expression, fasting glycemia, and hepatic insulin sensitivity (Sajan et al., 2015). In the present study, we found that the expression of PGC-1α and PEPCKE were positively correlated, which is consistent with previous research. PEPCK is the main rate limiting enzyme of gluconeogenesis, our results showed that the expression of PEPCK was increased in T2DM–NAFLD rat, contributing to hepatic gluconeogenesis, these changes can be attenuated by administration of bicyclol. These suggest that bicyclol plays anti-gluconeogenesis effects *via* regulating Akt/PGC-1α expression in insulin-resistant states.

Gluconeogenesis is the major pathway of endogenous glucose during fasting or starvation, the increase in the rate of hepatic gluconeogenesis will cause disorders in the regulation of glycogen, and then hyperglycemia. The current results showed that bicyclol decreased FBG levels in the rat with T2DM–NAFLD, indicating that bicyclol could anti-hyperglycemic. Liver is the transfer station of energy metabolism, excessive glycogen output will cause glycogen synthesis to turn into lipogenesis, causing abnormal liver lipid metabolism. Many metabolic intermediates were produced during pathological overnutrition, which oversaturate and constantly activate both hepatic lipogenesis and gluconeogenesis, whereas increased the hepatic lipid storage (Sun et al., 2012). It is clear from some studies (Hakimi et al., 2005) hepatic PGC-1α/PEPCK signaling play a key role in the liver lipid metabolism and PEPCK is required not only for gluconeogenesis but also for lipid accumulation and steatosis (Fang et al., 2020). These results once again proved the close relationship between gluconeogenesis and liver lipid metabolism. Hepatic steatosis is another major histopathological finding in T2DM–NAFLD rat model. Studies in our laboratory, bicyclol could significantly improve dyslipidemia and hepatic steatosis in T2DM–NAFLD rat, including the lowered TC, TG and intrahepatic fat accumulation. It suggests that the gluconeogenesis also plays an important role in liver glucose and lipid metabolism. However, due to the particularity of the T2DM disease, when the model was established, T2DM–NAFLD rat showed significant weight loss. Although the high-fat diet was maintained for a long time, it was still unavoidable. This made our experimental results biased, so we passed comparison the blank treatment group and the blank group found that the application of bicyclol alone can also reduce the weight of rats, which proves that bicyclol has a weight-reducing effect.

**FIGURE 8:**
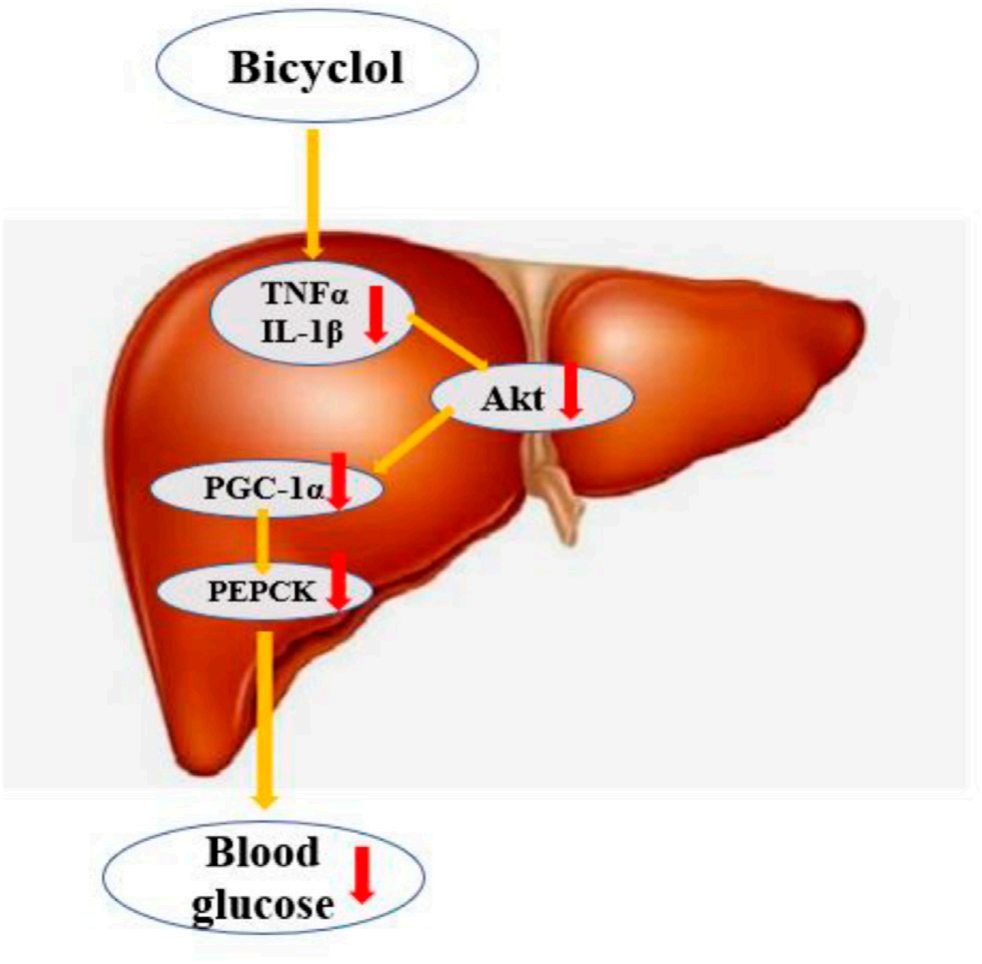
Bicyclol regulates hepatic gluconeogenicactivity mainly through inhibition inflammation and Akt/PGC-1α signal pathway.

In summary, the administration of bicyclol attenuates glycolipid toxicity of T2DM–NAFLD rat. Additionally, we examined the possible mechanisms and suggested that bicyclol may regulates hepatic gluconeogenic activity mainly through inhibiting inflammation *via* Akt/PGC-1α signal pathway (Figure 8). Therefore, our study reveals a novel regulatory network of bicyclol on liver gluconeogenesis, in which bicyclol could be a new therapeutic approach to treat T2DM–NAFLD and other metabolic disorders.

## Data Availability

The original contributions presented in the study are included in the article/Supplementary Material, further inquiries can be directed to the corresponding author.
